# Diversity and Distribution of Bryophytes Along an Altitudinal Gradient on Flores Island (Azores, Portugal)

**DOI:** 10.3390/plants14243766

**Published:** 2025-12-10

**Authors:** Rosalina Gabriel, Leila Nunes Morgado, Silvia Poponessi, Débora S. G. Henriques, Márcia C. M. Coelho, Gabriela M. Silveira, Paulo A. V. Borges

**Affiliations:** 1 University of the Azores, CE3C—Centre for Ecology, Evolution and Environmental Changes, Azorean Biodiversity Group, CHANGE—Global Change and Sustainability Institute, School of Agricultural and Environmental Sciences, Rua Capitão João d’Ávila, Pico da Urze, 9700-042 Angra do Heroísmo, Portugalpaulo.av.borges@uac.pt (P.A.V.B.); 2 Institute for Alpine Environment, Eurac Research, Viale Druso 1, 39100 Bolzano, Italy; silvia.poponessi@eurac.edu; 3 Banco Genético Vegetal Autóctone, Empresa Municipal Cascais Ambiente, Estrada de Vale de Cavalos, 2645-138 Cascais, Portugal; 4 University of the Azores, Gaspar Frutuoso Foundation, Rua Capitão João d’Ávila, Pico da Urze, São Pedro, 9700-042 Angra do Heroísmo, Portugal

**Keywords:** altitude, bryoflora, elevational gradient, endemic species, GIMS, liverworts, Macaronesia, mosses, substrate specificity, Wallacean shortfall

## Abstract

Altitudinal gradients offer powerful natural frameworks to investigate how environmental factors shape biodiversity, especially on young oceanic volcanic islands where short spatial distances encompass sharp climatic transitions. This study documents bryophyte diversity and examines how elevation, substrate, and environmental variables influence the structure of bryophyte communities on Flores Island (Azores). Across five sites and 385 microplots, 89 species from 37 families were recorded, with liverworts predominating (liverwort-to-moss ratio of 1.41). Species richness and abundance followed a unimodal pattern, peaking at mid-elevations (400–600 m a.s.l.), where humid and thermally stable conditions favor the coexistence of lowland and montane *taxa*. Even modest altitudinal shifts corresponded to pronounced turnover in community composition, revealing strong ecological filtering along the gradient. Substrate type further influenced diversity patterns, with liverworts dominating epiphytic and lignicolous habitats, while mosses were more diverse on terricolous and rupicolous substrates. The presence of several Azorean and Macaronesian endemics, including threatened *taxa*, highlights the conservation importance of mid-elevation habitats. Overall, these results show that fine-scale altitudinal variation generates substantial ecological differentiation, underscoring the role of montane forests as refugia for hygrophilous and endemic bryophytes and as sensitive indicators of environmental change in island ecosystems.

## 1. Introduction

Islands represent valuable natural laboratories for advancing the theoretical understanding of ecological processes, due to their geographic isolation and the distinctive composition of their biota [1]. Oceanic islands originate from submarine volcanic activity, predominantly characterized by basaltic substrates and remaining geologically isolated from continental landmasses. Their biotic assemblages were initially established through long-distance dispersal, followed by in situ speciation, which contributed to the development of unique evolutionary trajectories [2]. Generally, oceanic islands have lower species richness per unit area than equivalent continental regions [2] but demonstrate higher levels of endemism [3,4].

Thus, the biogeographical patterns observed on oceanic islands are shaped by a complex interplay of factors operating across multiple spatial and temporal scales, influencing species’ richness, endemism [5,6], and extinction dynamics [7]. Likewise, altitudinal patterns of species richness result from the combined effects of climatic, biological, geographical, and historical factors [8,9] as demonstrated in Macaronesian Islands [10]. Typically, but not always, species richness decreases with increasing altitude, often showing a mid-elevation peak where environmental heterogeneity is greatest [9,11,12]. Hence, altitude is frequently used as a proxy for multiple biotic and abiotic gradients that together shape the composition and structure of ecological communities [13].

Studies on the diversity and distribution of vascular plants and bryophytes along elevation gradients have been conducted in different island ecosystems. For vascular plant species, Tassin and colleagues [14] showed that the species richness of the native woody flora on Réunion Island (Mascarene Archipelago, Indian Ocean) increased up to 700–750 m a.s.l. and declines above 1000 m a.s.l., whereas endemicity increases linearly with altitude. Also, on Réunion Island, Ah-Peng and colleagues [15], analyzed bryophyte diversity along an altitudinal transect (250–850 m a.s.l.) and found that total bryophyte richness (comprising 55 liverworts, one hornwort, and 14 mosses) increased with elevation, with liverwort richness contributing strongly to this pattern. Similarly, an analysis of epiphytic bryophytes along a wider elevation gradient on Réunion (350–2750 m a.s.l.), found a peak in species richness at mid-elevations [11]. On Sicily, a continental island, bryophyte richness also follows a hump-shaped relationship with the elevation, with a peak at 1200–1700 m a.s.l. [16]. Beyond individual island studies, a recent global synthesis by Maul and colleagues [17] showed that liverworts exhibit a consistent mid-elevation richness peak across most mountain ranges worldwide. Relative elevation was identified as the strongest predictor of richness, suggesting that the mixing of low- and high-elevation floras along steep ecological gradients drives this pattern. Climatic variables related to temperature and water availability further explained variation in richness, indicating that low-elevation liverwort communities may be especially susceptible to warming and increasing drought.

In the Azores archipelago, bryophyte diversity along altitudinal gradients was studied in Pico [12,18] and Terceira islands [19,20,21], using protocols comparable to those applied in La Réunion [11,22,23]. On both islands, species’ richness peaks between 600 and 1000 m a.s.l. The patterns observed on Pico and Terceira are broadly similar within their shared elevation range; the main difference is that Pico extends to much higher elevations. Above 1000 m a.s.l., richness on Pico declines sharply toward the summit at 2200 m a.s.l. [12,19], whereas on Terceira, which reaches only 1000 m, richness remains relatively stable up to the island’s highest elevations, with only a slight decline near the top [20,21].

Pico and Terceira are arguably the best-preserved islands of the Azores, a region where most natural vegetation has been altered or degraded due to anthropogenic activities, particularly at low-middle elevations, with native forests experiencing the most significant impact [23,24]. Well-preserved vegetation remnants are found at elevations above 600 m a.s.l., primarily consisting of *Juniperus*-*Ilex* forests and *Juniperus* woodlands [25].

Flores island, a much smaller (143 km^2^), and isolated island, located in the American tectonic plate, displays a humid oceanic climate characterized by significant orographic precipitation [26], making it an optimal environment for bryophyte diversity [27]. Although not reaching 1000 m a.s.l., the distribution of bryophyte species in Flores Island is distinctive, as studied by the Allorge couple after their visit in 1937 [27]. At the time, the richest strata were associated with forests, which in Flores are found at lower altitudes than is typically found in the archipelago. This is probably due to the highest levels of precipitation, which creates favorable conditions for the establishment of these forests below 700 m a.s.l. [25]. Presently, the island still harbors remnant native forests, which constitute an important habitat for bryophytes, including Macaronesian endemics, which have restricted distributions.

Due to its isolation, well-marked elevational range, and relatively undisturbed habitats, Flores offers a valuable natural laboratory for studying bryophyte diversity and distribution along altitudinal gradients. Despite this potential, no systematic study has yet assessed bryophyte richness in relation to altitude on this island. Understanding how elevation shapes patterns of bryophyte richness is therefore essential for clarifying the ecological mechanisms underlying their diversity and spatial distribution.

The aim of this study is to (1) document bryophyte species richness, composition, and colonization status along an elevation gradient on Flores Island, and (2) evaluate how substrate type contributes to variation in bryophyte diversity and community structure.

## 2. Results

### 2.1. Species Inventory and Sampling Completeness

#### 2.1.1. Floristics

**Figure 1:**
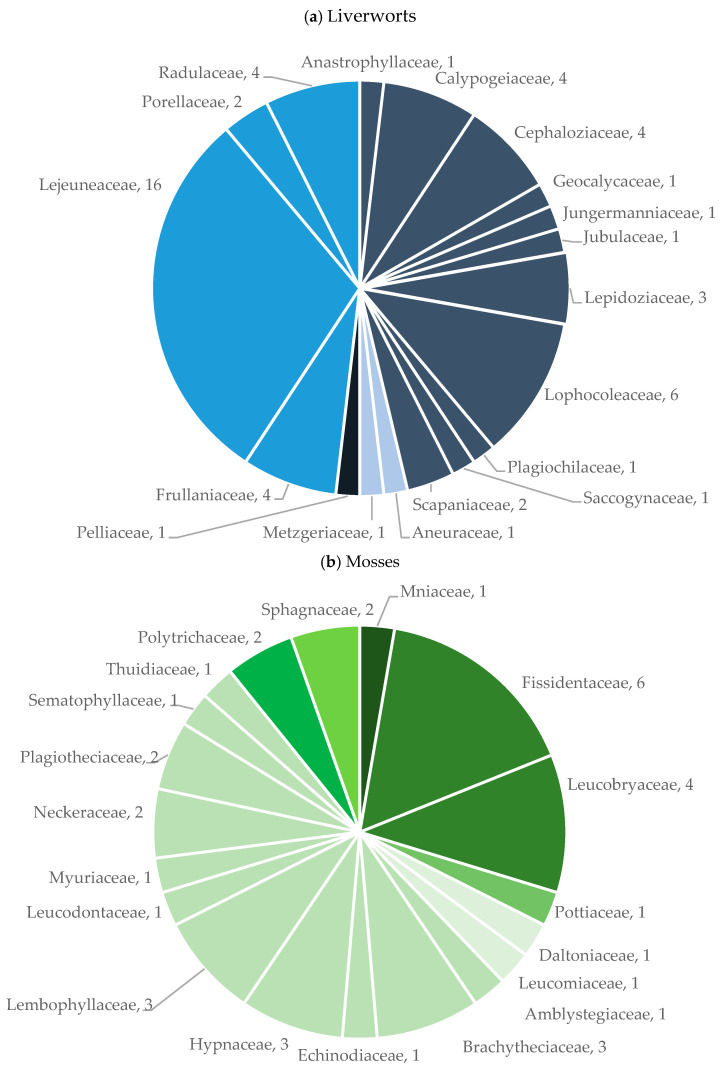
(**a**) Number of species per family within liverworts. Each slice represents a family, with size proportional to species richness. Colors correspond to the Order of each Family: Jungermanniales, Metzgeriales, Pelliales, and Porellales. (**b**) Number of species per family within mosses. Each slice represents a family, with size proportional to species richness. Colors indicate the Order of each Family: Bryales, Dicranales, Pottiales, Hookeriales, Hypnales, Polytrichales, and Sphagnales.

Five localities were surveyed along the elevation gradient of Flores Island, distributed between 70 and 800 m a.s.l. (200 m elevation steps). In total, 385 microplots were sampled, resulting in the identification of 37 families (19 Bryophyta and 18 Marchantiophyta), 57 genera (27 Bryophyta and 30 Marchantiophyta), and 89 species (37 Bryophyta and 52 Marchantiophyta) [28,29] (Figure 1). The overall ratio of liverwort-to-moss was 1.41. The Family Lejeuneaceae was the most diverse, with 16 species, followed by families Lophocoleaceae and Fissidentaceae, each represented by six species. Regarding abundance, Lejeuneaceae was also the most abundant family, comprising 316 of the 1345 specimens collected (24%), then followed by Frullaniaceae (*n* = 201; 15%), Radulaceae (*n* = 106; 8%), Hypnaceae (*n* = 78; 6%), and Fissidentaceae (*n* = 71; 5%), while all remaining families accounted for ≤70 specimens (≤5%). At the generic level, the most representative *taxa* were *Frullania* (*n* = 202; 15%), *Lejeunea* (*n* = 130; 10%), *Radula* (*n* = 106; 8%), *Hypnum* (*n* = 77; 6%), and *Fissidens* (*n* = 71; 5%) with all other genera represented by 70 specimens or less (5%). Owing to the scarcity of material, particularly among epiphylls, three specimens could only be assigned to family, and 68 to the genus level.

At the sample level, the ten most frequently recorded species included six liverworts (*Frullania acicularis*, *F. microphylla*, *Marchesinia mackaii*, *Lejeunea eckloniana*, *Radula carringtonii*, *Drepanolejeunea hamatifolia*) and four mosses (*Hypnum uncinulatum*, *Pseudotaxiphyllum laetevirens*, *Fissidens taxifolius* and *Echinodium renauldii*).

Six liverwort species (*Frullania acicularis*, *F. microphylla*, *Lejeunea eckloniana*, *Lejeunea hibernica*, *L. lamacerina*, *Saccogyna viticulosa*), but no moss species were found in encompassing the whole altitudinal gradient, with *Campylopus flexuosus* and *Hypnum uncinulatum* present in four of the five surveyed levels, from 200 to 800 m a.s.l. A higher number of species (17 mosses, 17 liverworts), were only found in one elevation, and about a fifth of the species were only recorded once in the gradient (*N_uniques_* = 18; 13.2%) (e.g., *Exsertotheca crispa*, *Isopterygiopsis pulchella*, *Tetrastichium virens*, *Fuscocephaloziopsis lunulifolia*).

With respect to substrate, most liverwort species (*n* = 42; 81%) occurred on trees, whereas fewer than half of the moss species (*n* = 16; 43%) were found on that substrate.

#### 2.1.2. Colonization Status and Vulnerability

Four Azorean endemic *taxa* were recorded in the present survey (Table S1): *Leptoscyphus porphyrius* subsp. *azoricus*, *Fissidens azoricus*, *Rhynchostegiella azorica*, and *Echinodium renauldii* [30,31]. Among these, *F. azoricus* is classified as Critically Endangered under the IUCN Red List criteria; the pleurocarpous moss *E. renauldii* and the foliose liverwort *L. porphyrius* subsp. *azoricus* are both considered as Endangered, whereas *Rhynchostegiella azorica* is listed as Near Threatened [32]. Seven Macaronesian endemic species were also recorded (Table S1). These include the liverwort *Calypogeia azorica* (Endangered), and two pleurocarpous mosses, *Andoa berthelotian* and *Isothecium prolixum*, both assessed as Vulnerable. The remaining four Macaronesian endemics are classified as Near Threatened. In addition, twelve European endemic species were found, with four listed as Near Threatened and eight as Least Concern. The remaining *taxa* (*n* = 66) are regarded as native; among these, most (*n* = 56) are assessed as Least Concern, five as Near Threatened, two as Vulnerable (*Cololejeunea azorica*, *Lejeunea mandonii*), and one as Endangered (*Cololejeunea sintenisii*). Two species have not yet been evaluated. No invasive bryophyte species were recorded on this survey.

#### 2.1.3. Sampling Completeness

Sampling completeness, estimated with the first-order jackknife, reached 83% for the overall bryophyte assemblage, with a higher value for liverworts (88%; 52 species) compared to mosses (77%; 37 species). The lower completeness value obtained for mosses reflects the higher proportion of unique or infrequent species in this group; nevertheless, the estimated coverage is still adequate to provide a reliable basis for further studies of the sampled communities.

### 2.2. Altitudinal Gradient and Mid-Domain Effect

**Figure 2:**
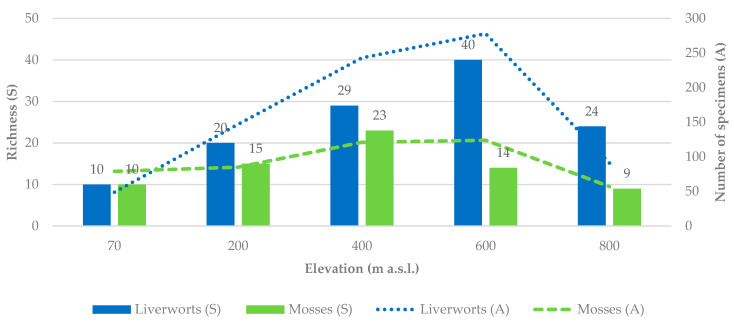
Bryophyte (liverworts and mosses) richness and abundance (number of specimens present in 5 cm × 10 cm microplots; see the secondary axis) along Flores Island’s elevational gradient.

Bryophyte richness (S) increased with altitude up to mid-elevations and declined towards the uppermost site, forming a clear unimodal pattern (Figure 2).

The highest values were recorded at 400 m a.s.l. (*S* = 52) and 600 m a.s.l. (*S* = 54), indicating that mid-elevations support both greater abundance and diversity of bryophytes. Similarly, the number of identified specimens increased from the lowest altitude sampled (70 m a.s.l.), reaching a peak at 600 m a.s.l. (*n* = 402; 31.65%), before declining at 800 m a.s.l. (*n* = 147; 11.57%). Overall, the sites at mid-elevation accounted for approximately 60% of all recorded specimens and species.

The null model predicted a peak at 400–600 m, consistent with the expectation of the mid-domain effect. Observed richness values were always higher than the simulated mean, falling outside of the 95% confidence intervals. However, the Spearman correlation between observed and predicted richness was strong and significant (ρ = 0.95, *p* = 0.014), indicating that the overall shape of the richness–elevation relationship closely matched that expected under random geometric constraints.

**Figure 3:**
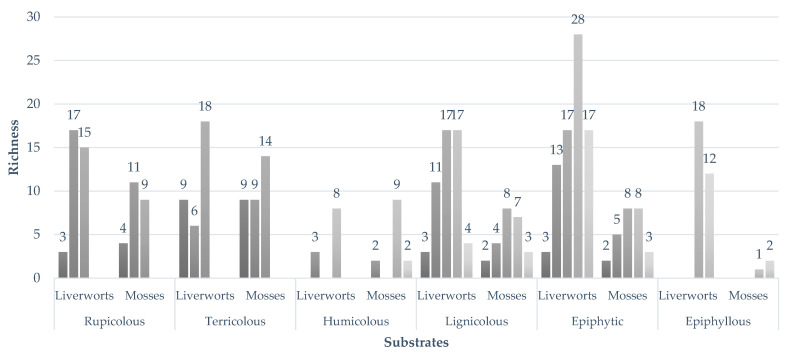
Richness of liverworts and mosses recorded across substrates and elevations on Flores Island. Bars show the number of species in each substrate type (rupicolous, terricolous, humicolous, lignicolous, epiphytic and epiphyllous) at the five elevational bands (70, 200, 400, 600 and 800 m a.s.l.) (see also Section 2.3).

The number of sampled substrates varied across elevations, reflecting differences in substrate availability along the gradient. Nonetheless, all elevational bands included a substantial sampling effort (59–60 samples at the lowest and highest sites and 84–92 at the intermediate elevations) and included both ground-based substrates (e.g., rupicolous, terricolous, humicolous) and plant-based substrates (e.g., lignicolous, epiphytic, and epiphyllous). This ensured consistent representation of the main bryophyte microhabitats at each site and allowed the assessment of how species richness changes with elevation and substrate (Figure 3).

**Figure 4:**
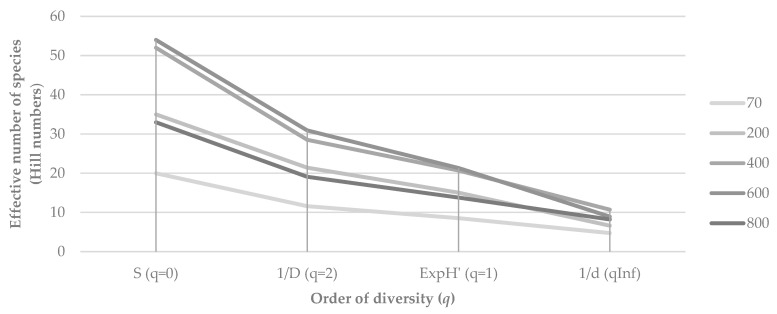
Hill diversity profiles of bryophyte communities along an elevational gradient on Flores Island (Azores).

To complement the observed patterns in abundance and species richness, we assessed α-diversity using Hill numbers, which integrate information on both species’ richness and relative abundance on Flores Island (Figure 4). Species richness (*q* = 0) and effective numbers of common and dominant species (*q* = 1–2) increased from low elevations (70 m) to mid-elevations (400–600 m), where diversity reached its maximum. At the highest altitude (800 m), all diversity measures declined markedly.

The decreasing difference between H_0_, H_1_ and H_2_ at mid-elevations suggests greater evenness in species abundances, whereas wider gaps at the extremes of the gradient (70 m and 800 m) point to stronger dominance by a few *taxa*. Overall, the results suggest that bryophyte α-diversity on Flores Island peaks under intermediate environmental conditions.

**Figure 5:**
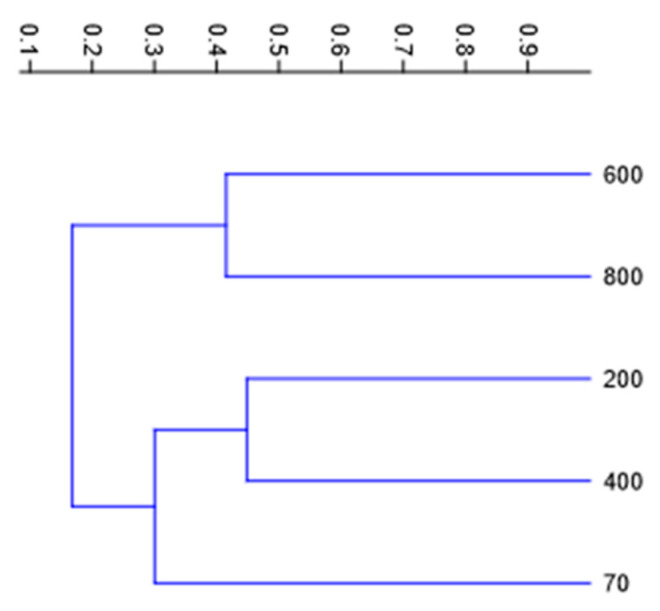
UPGMA dendrogram showing the similarity relationships among bryophyte assemblages sampled at five elevations (70 m, 200 m, 400 m, 600 m, and 800 m) on Flores Island.

To explore whether these variations in diversity and evenness correspond to differences in community composition along the gradient, we conducted a clustering analysis. The UPGMA dendrogram, based on Bray–Curtis distances, revealed a clear altitudinal structuring of bryophyte assemblages on Flores Island (Figure 5).

Samples from low to mid elevations (70 m, 200 m, and 400 m) clustered together at relatively low dissimilarity values, indicating broadly similar community composition along this altitudinal range. In contrast, the high-elevation sites (600 m and 800 m) formed a separate and internally coherent cluster, reflecting a distinct bryophyte assemblage characteristic of upper montane conditions. The higher Bray–Curtis distances separating the two major clusters suggest a marked compositional shift between mid- and high-elevation communities, likely associated with environmental gradients such as increasing humidity, lower temperatures and greater cloud frequency at higher altitudes.

### 2.3. Substrate Specificity of Bryophyte Assemblages

**Figure 6:**
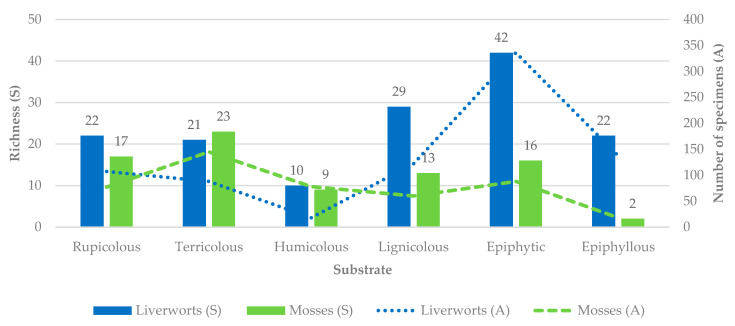
Bryophyte (liverworts and mosses) richness and abundance (number of specimens present in 5 cm × 10 cm microplots; see the secondary axis) along six substrates on Flores Island.

The number of species and specimens varies according to the different substrates sampled. Figure 6 illustrates the variation in species richness of liverworts and mosses across different substrate types on Flores Island. Epiphytic and lignicolous substrates supported the highest numbers of liverwort species (42 and 29, respectively), whereas mosses were most diverse on terricolous (23) and rupicolous (17) substrates. In contrast, epiphyllous and humicolous substrates harbored fewer species of both groups.

**Figure 7:**
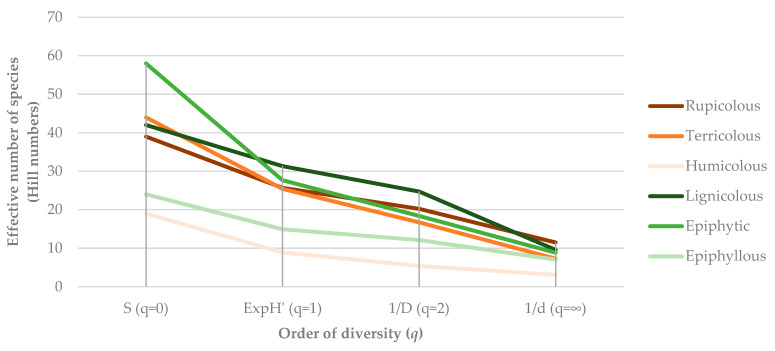
Hill diversity profiles of bryophyte communities across substrate types on Flores Island (Azores).

Consistent with the results for species richness and abundance, the analysis of α-diversity based on Hill numbers (H) reveals similar patterns across bryophyte communities (Figure 7). Species richness (*q* = 0) was highest on epiphytic (58 spp.), terricolous (44 spp.) and rupicolous (39 spp.) substrates, and lowest on humicolous (19 spp.) and epiphyllous (24 spp.) ones. When abundance was incorporated, both the effective number of species (ExpH’, *q* = 1) and Simpson diversity (*q* = 2) showed the same general trend: lignicolous and epiphytic communities exhibited the highest diversity and evenness, while humicolous assemblages were clearly less diverse and strongly dominated by a few species, namely *Sphagnum palustre*. 

Differences between H_0_, H_1_ and H_2_ were smallest in lignicolous and epiphytic bryophyte assemblages, indicating relatively even species distributions and balanced dominance structures. In contrast, the wider gaps observed in humicolous and epiphyllous communities reflect stronger dominance by a few *taxa* and a high proportion of rare species.

Overall, these results show that substrate type exerts a strong influence on bryophyte α-diversity on Flores Island. Lignicolous and epiphytic habitats provide a wide range of microclimatic and structural niches that support diverse and compositionally balanced communities, whereas humicolous and epiphyllous substrates represent more restrictive or transient environments, supporting fewer and more unevenly distributed species.

**Figure 8:**
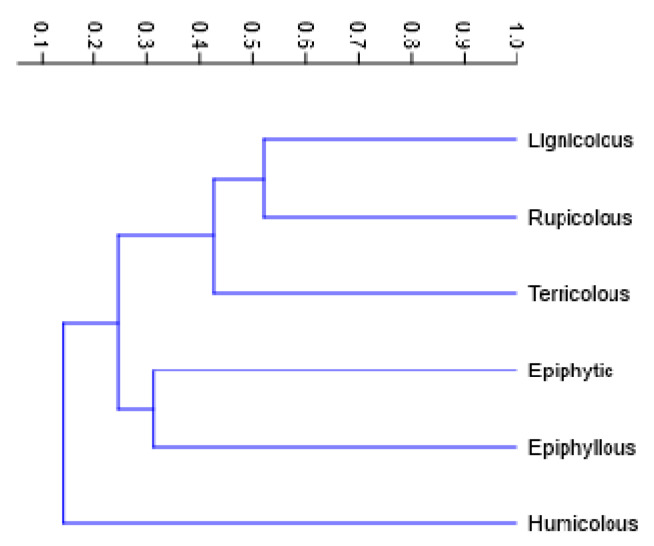
UPGMA dendrogram showing similarity relationships among bryophyte assemblages associated with six substrate types (rupicolous, terricolous, humicolous, lignicolous, epiphytic, and epiphyllous) on Flores Island.

Given the marked variation in species richness and evenness across substrate types, a UPGMA clustering analysis based on Bray–Curtis dissimilarities was conducted to examine the similarity relationships among the bryophyte assemblages (Figure 8).

The dendrogram reveals two major clusters. Lignicolous, rupicolous, and terricolous assemblages, form a cohesive “ground-substrate”, joining at relatively low dissimilarity values (similarity ≈ 0.4–0.5), suggesting that these substrates share a subset of species. Epiphytic and epiphyllous assemblages cluster together at moderate similarity levels (similarity ≈ 0.3–0.4).

In contrast, humicolous communities form a clearly distinct branch, exhibiting low similarity with all other substrate types (≈0.1–0.2). This separation indicates that bryophyte assemblages associated with soil or decaying organic matter are compositionally unique, likely driven by specific microclimatic and nutrient conditions. Overall, the clustering pattern highlights substrate specificity as a key of bryophyte community structure.

## 3. Discussion

### 3.1. General Overview and Sampling Completeness

This study provides a structured and up-to-date field survey of bryophytes along an elevational gradient on Flores Island, complementing the pioneering works the Allorge couple and Persson in 1937 [27] and Erik Sjögren in 1993 [33]. It documents 89 species across 37 families and 57 genera, with a sampling completeness of 83%. Although this represents only about one third (33.8%) of the bryophyte flora previously reported for the Island and circa a fifth (18%) of the Azores archipelago bryophyte diversity [30,31], these values are notable given the small spatial extent of our plots and the forest-focused design of the study. As in Pico Island [12], several species were new records for the surveyed sites, especially at lower elevations. Rather than aiming at exhaustive floristic coverage, the goal was to characterize fine-scale ecological patterns, and in this context, the stratified GIMS protocol [34], which incorporates multiple elevation levels and six substrate types, proved effective for capturing a meaningful subset of the island’s regional bryophyte diversity. Species not detected are likely associated with habitats that fall outside the scope of our forest-based sampling (e.g., bogs, stream margins, urban areas, grasslands, or mixed exotic woodlands), suggesting that the true alpha-diversity of Flores may still be under-represented in existing floristic inventories.

The observed ratio of liverworts to mosses (1.41) and the predominance of leafy liverwort families such as Lejeuneaceae, Frullaniaceae, and Radulaceae is characteristic of oceanic, hyper-humid environments [35,36,37] and confirms earlier descriptions of Azorean bryophyte assemblages as highly hygrophilous and dominated by liverworts [12,19,20,21,29,38,39,40,41]. The higher completeness for liverworts (88%) compared to mosses (77%) may reflect the ecological dominance of epiphyllous and epiphytic species on Flores Island. Despite this, the estimated coverage is sufficient to support robust ecological inferences, capturing the main floristic and functional composition of the island’s bryophyte communities.

These results indicate that Flores retains well-preserved humid forest habitats, especially at mid- and upper-elevation zones, and are consistent with patterns observed on other Azorean islands, where montane bryophyte assemblages are dominated by oceanic liverworts and pleurocarpous mosses adapted to shaded, moisture-saturated conditions [12,27,39]. The presence of four Azorean endemic *taxa*, including the conservation concern species *Fissidens azoricus* and *Echinodium renauldii*, further highlights the biogeographical distinctiveness of the bryoflora of Flores and its contribution to the regional pool of Macaronesian endemics, reinforcing the role of natural areas as refugia, areas where species can retreat to, persist under changing environmental conditions and potentially expand from [42]. Previous assessments of climate-related threats to the Macaronesian endemic flora indicate that such refugial areas will become increasingly critical in the coming decades, as projections suggest an average decline of more than 50% in suitable habitat per species [43,44].

### 3.2. Altitudinal Gradients of Diversity and Compositional Turnover

Along the elevational transect, bryophyte richness and abundance exhibited a unimodal pattern, with a peak at mid-elevations (400–600 m a.s.l.), consistent with the intermediate productivity and environmental heterogeneity hypotheses commonly described for island ecosystems. Because elevation on oceanic islands is bounded by sea level below and the mountain summit above, the ranges of low- and high-elevation *taxa* necessarily overlap toward the center of the gradient, producing a tendency toward increased richness at mid elevations. This mid-elevation maximum has been documented in other insular and montane ecosystems and is often attributed to the convergence of favorable climatic and structural conditions, including high atmospheric humidity, moderate temperatures, and increased substrate heterogeneity [9,13,15,45,46]. This mechanism is also consistent with the global synthesis of Maul and colleagues [17], who showed that the relative position along the gradient is a strong predictor of liverwort richness, reflecting the admixture of floras and the transition between contrasting environments. At the same time, the consistently higher observed richness relative to geometric predictions indicates that environmental factors, such as substrate diversity, humidity, or microclimatic stability, amplify this mid-elevation peak. Mid-elevations on Flores correspond to persistent cloud cover, characterized by stable humidity, mild temperatures, and heterogeneous substrates, conditions particularly favorable for bryophyte establishment and persistence.

The combined geometric and environmental processes provide a coherent explanation for the observed pattern. In fact, at low elevations (70–200 m), reduced humidity, higher temperatures, and greater exposure to wind and salt spray may limit bryophyte colonization and survival, favoring a smaller subset of stress-tolerant species. On the other hand, at high elevations (800 m), decreasing temperature and reduced substrate diversity, with the ground dominated by *Sphagnum palustre*, may constrain species establishment, leading to a decline in both richness and evenness. Such a pattern aligns with previous studies from Macaronesia showing similar unimodal responses of bryophyte diversity to elevation [12,47,48].

Community clustering analyses reinforce this interpretation by showing two major assemblage groups: one comprising the low-to-mid elevations (70–400 m) and another representing the upper montane sites (600–800 m). This compositional turnover suggests the existence of distinct bryophyte communities structured primarily by altitudinal microclimates and associated vegetation zones. Such elevational differentiation reflects both environmental filtering and limited dispersal across sharp humidity and temperature gradients typical of oceanic islands.

### 3.3. Substrate Specificity and Ecological Differentiation

Substrate type proved to be a key determinant of bryophyte assemblage composition and diversity. Epiphytic and lignicolous substrates supported the highest numbers of liverwort species, reflecting the importance of tree bark and decaying wood as stable, moisture-retaining habitats that buffer microclimatic fluctuations. These substrates are characterized by surface roughness, organic matter accumulation, and capillary water retention, conditions that favor species with thin, delicate gametophytes such as members of Lejeuneaceae and Frullaniaceae [49]. By contrast, terricolous substrates harbored the highest proportion of mosses. The lowest total diversity was found in humicolous communities, greatly dominated by *Sphagnum palustre* and *Polytrichum commune*, and epiphyllous communities, which represent more transient or environmentally unstable habitats. These results are consistent with those observed in other oceanic and subtropical islands, where substrate heterogeneity has been shown to be a critical driver of bryophyte α-diversity [50,51].

Differences in evenness across substrates, as revealed by Hill numbers, further highlight how substrate-specific microclimates influence community structure. Lignicolous and epiphytic assemblages exhibited relatively even species distributions, while humicolous and epiphyllous habitats were dominated by a few dominant species. This supports the view that habitat heterogeneity and substrate stability underpin both local and regional bryophyte diversity, especially on islands where spatial turnover is constrained by limited area and dispersal barriers.

### 3.4. Biogeographical and Conservation Implications

Taken together, the patterns observed across elevation and substrate types indicate that diversity on Flores Island, as in other islands and archipelagos [52,53], is structured by the interaction of climatic gradients and microhabitat heterogeneity. The strong correspondence between community composition and elevation suggests that temperature, humidity, and forest structure act as key filters determining species occurrence. However, the influence of substrate type shows that local-scale factors also mediate these effects, reinforcing the importance of fine-scale environmental complexity in sustaining high bryophyte richness on oceanic islands.

The floristic composition of Flores Island also reveals the coexistence of widespread temperate bryophytes including Azorean and Macaronesian endemics, several of which are threatened. The occurrence of *Fissidens azoricus*, *Leptoscyphus porphyrius* subsp. *azoricus*, and *Echinodium renauldii*, classified as Critically Endangered or Endangered under IUCN criteria [32], emphasizes the conservation importance of maintaining humid montane forest habitats. These environments, concentrated around mid-elevations, coincide with the areas of highest bryophyte richness identified in this study, reinforcing their value as refugial ecosystems under changing climatic regimes.

Given the small area of Flores Island, the strong elevational structuring detected here suggests that even modest shifts in climatic zones may have disproportionate impacts on bryophyte distributions. Species restricted to narrow elevational belts may face habitat contraction or local extinction if suitable microclimates shift upward [43,44] or downwards [44]. Maintaining habitat continuity across the elevational gradient, particularly through the conservation of native forest remnants and the restoration of promising fragments such as the site on 400 m elevation, is therefore crucial to facilitate altitudinal migration and preserve community connectivity.

At a regional scale, the findings contribute to the growing understanding of how island topography, microclimate, and habitat diversity interact to shape bryophyte biogeography in Macaronesia. The predominance of oceanic liverworts and the strong elevational signal suggest that Flores still retains conditions resembling pre-disturbance montane forests. However, anthropogenic alterations, such as the establishment of invasive species, habitat fragmentation and disruption of humidity gradients may hinder the diversity and conservation of bryophytes in the Azores and Macaronesia.

### 3.5. Limitations and Perspectives

While this study provides robust evidence of the main drivers of bryophyte diversity on Flores, and demonstrates the effectiveness of the GIMS protocol [34], derived from the modified BRYOLAT method [11,22], for surveying bryophyte communities across elevation gradients, future research could benefit from incorporating finer-scale microclimatic measurements (e.g., substrate moisture, canopy cover, irradiance, evapotranspiration) and temporal replication to capture seasonal variability. This would be especially important for *taxa* identifiable only during a narrow phenological window, such as shuttle species, in the Family Ricciaceae.

Examining additional dimensions of biodiversity, including functional and genetic diversity, would help explain ecosystem functioning, stability, and resilience than species richness alone. Integrating molecular approaches could also improve taxonomic resolution for morphologically cryptic *taxa* and enable the detection of intraspecific responses to elevation.

Moreover, long-term monitoring along the same altitudinal transect would be essential for assessing the sensitivity of bryophyte communities to ongoing climate change, especially in mid-elevation zones that appear both species-rich and potentially vulnerable. Establishing one or more additional altitudinal transects elsewhere on the island would further increase statistical power and strengthen the generality of elevation-related patterns.

## 4. Methodology

### 4.1. Study Area

#### 4.1.1. Azores Archipelago

The Azores archipelago consists of nine oceanic islands of volcanic origin, situated in the North Atlantic Ocean, between 36°55′ and 39°43′ N latitude and 25°00′ and 31°17′ W longitude, approximately 1600 km from the western coast of the European continent and nearly 2000 km away from North America [54].

**Figure 9:**
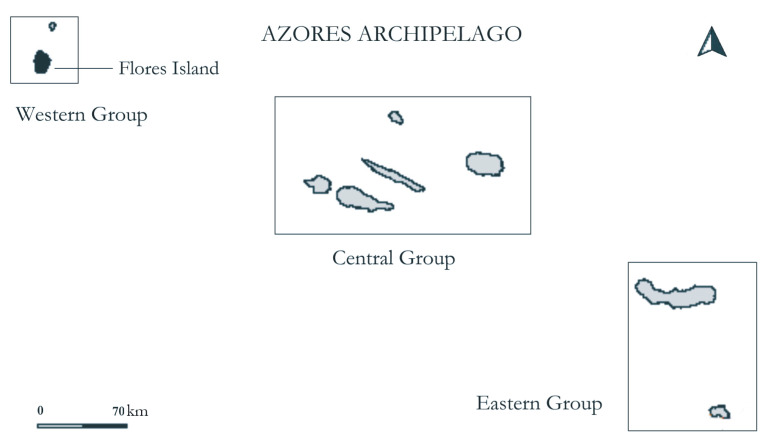
Map of the Azores archipelago, composed of nine volcanic islands in the North Atlantic. The study was conducted on Flores Island, shown in black.

Geographically, the archipelago is divided into three island groups: The Western Group (Flores and Corvo) located on the North American tectonic plate, the Central Group (Pico, Faial, São Jorge, Terceira, and Graciosa), and the Eastern Group (São Miguel and Santa Maria), both located on the Eurasian tectonic plate (Figure 9). The oldest island in the Azores archipelago is Santa Maria, estimated to be 6.01 million years old [55], while the youngest is Pico Island, approximately 0.186 million years old [56].

The Azores archipelago encompasses a total land area of 2323 km^2^, with Corvo (17 km^2^) being the smallest island, São Miguel (745 km^2^) the largest, and Flores Island being the fourth smallest (143.1 km^2^). Pico Island is the highest (2350 m a.s.l.), and only four other islands reach more than 1000 m a.s.l. (São Miguel, 1105 m a.s.l.; São Jorge, 1053 m a.s.l.; Faial, 1043 m a.s.l.; Terceira, 1021 m a.s.l.), while Flores Island peaks at 915 m a.s.l. in *Morro Alto* [54].

Due to its geographical location, the Azores exhibit a predominantly maritime climate, characterized by mild temperatures with a narrow thermal amplitude, high relative humidity, and significant precipitation during autumn and winter [57]. The mean annual temperature ranges from 12 °C to 23 °C, while relative humidity remains consistently high across all seasons, exceeding 95% on more than 50 days per year, particularly in March, June, and December; annual precipitation at sea level varies between 1000 mm in the Eastern Group (particularly in Santa Maria Island) and 1600 mm in the western group [58]. Wind and precipitation are the primary climatic factors influencing local vegetation [59]. At high altitudes, wind speeds can exceed 100 km/h, accompanied by temperatures ranging from 0 °C to 5 °C, while precipitation, including horizontal precipitation, plays a crucial role in increasing the water availability in biological systems, promoting the growth of altitudinal vegetation through the Föhn effect [59,60,61].

Biogeographically part of Macaronesia, the Azores archipelago presents high biological and ecological interest in fauna and flora [62], and a remarkable socio-economic and cultural importance [63].

#### 4.1.2. Flores Island

Flores Island is located in the North Atlantic Ocean (between latitudes 36.9° N–39.7° N and longitudes 24.9° W–31.3° W) [54]. The island is thought to be 2.0 to 2.5 million years old [56] and its most recent volcanic activity occurred in the 15th century, already during the period of Portuguese colonization [64]. This volcanic phase was characterized by monogenetic, effusive, and explosive basaltic eruptions, which formed several scoria cones, maars, and tuff rings across the central area of the island [26,65]. The central area currently hosts the largest peat bog in the Region, which plays a vital role in regulating the island’s hydrological balance and contributes significantly to the formation of streams and waterfalls [66].

The Island benefits from a temperate oceanic climate, largely influenced by its development, orientation, and the presence of the Azores anticyclone. Consequently, the island’s landscape displays a variety of green shades, resulting from the high humidity levels and frequent natural landslides, which contribute to floristic and structural diversity, with extensive areas of pastures and woodlands [66,67].

Flores Island is administratively divided into two municipalities: *Santa Cruz das Flores* in the north and *Lajes das Flores* in the south. It has a resident population of 3.429 inhabitants, making it the second least populous island in the archipelago, with just 2.57% population of the most populated island São Miguel (~133.390 inhabitants) [68].

In 2009, the island was designated a UNESCO Biosphere Reserve, encompassing not only the entire emerged area of the island but also an adjacent marine zone, covering a total of 58.619 hectares; this mention recognizes the island’s unique landscape, geological, environmental, and cultural values at regional, national, and international levels [65].

#### 4.1.3. Sampling Design and Fieldwork Procedures

The methodology was designed to investigate the diversity and distribution of bryophytes along altitudinal gradients, with the aim of obtaining data on the environmental factors and substrates associated with the bryophyte community and roughly followed the BRYOLAT protocol [11], adapted for the Azores [22], later published as a Global Island Monitoring Scheme—GIMS [53].

The study was conducted between 29 July and 1 August 2013 in the municipality of Santa Cruz das Flores. Sampling sites differed in location, elevation (m a.s.l.), and geographic coordinates (decimal Latitude and Longitude) (Table 1).

**Figure 10:**
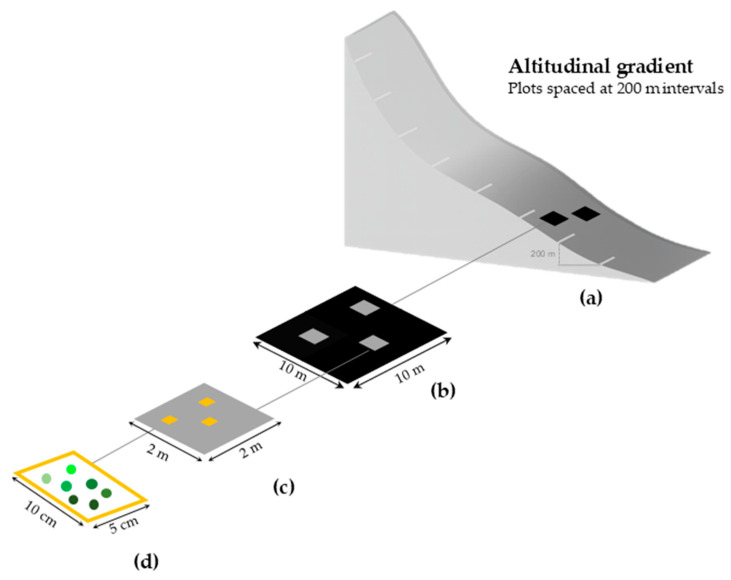
Sampling design used for bryophyte surveys along the altitudinal gradient on Flores Island, following the GIMS protocol based on the BRYOLAT methodology [11,18,21]. (**a**) Sampling was conducted at 200 m elevation intervals; at each elevation level, two plots (black squares, 10 m × 10 m) were established 10–15 m apart. (**b**) Each plot was subdivided into 25 quadrats (gray squares, 2 m × 2 m), of which three were randomly selected for sampling. (**c**,**d**) Within each selected quadrat, all available microhabitats were examined (e.g., soil, rock, bark, litter, decaying wood), and three microplots (yellow rectangles, 5 cm × 10 cm) were sampled per microhabitat. On trees, nine microplots were sampled across three height levels (1–50 cm, 51–100 cm; 101–200 cm).

Sampling plots were established in environmentally homogeneous areas that were representative of the best-preserved native vegetation fragments at each selected elevation (70, 200, 400, 600, and 800 m a.s.l.) (Figure 10). Although a linear arrangement of plots would have been preferable, this was not feasible due to constraints related to the spatial distribution of native vegetation and local topography, which in some cases (e.g., 400 m) prevented the safe and practical installation of plots along a straight transect.

Bryophyte specimens were collected by experienced bryologists, with particular care taken to minimize disturbance to the habitat and to avoid excessive removal of material from natural populations.

Bryophytes were sampled along the elevation gradient using the GIMS protocol for bryophytes [53] a modified BRYOLAT standardized method [11,22] in order to include taxonomic information on bryoflora and environmental data. At each site, two plots of 10 m × 10 m were set 10–15 m apart. Each plot was divided into 25 quadrats (2 m × 2 m), from which three were randomly selected for further inspection. Each quadrat was carefully examined to collect three microplots of 50 cm^2^ (10 cm × 5 cm) for all the substrates colonized by bryophytes: rock, soil, humus, organic matter, tree bark, and leaves/fronds, using the following nomenclatures: Rupicolous (RU); Terricolous (TE); Humicolous (HU); Lignicolous (LI); Epiphytic (T); Epiphyllous (LF). The distinction between terricolous and humicolous substrates was based on the dominant material directly underlying each microplot: terricolous samples showed a visible mineral component, whereas humicolous samples consisted mainly of organic remnants, mostly degraded *Sphagnum* material, with no apparent mineral structure. On trees, nine microplots were sampled across three height levels, lowest (1–50 cm), medium (51–100 cm), and higher (101–200 cm) levels [11,22,53].

Immediately following collection, microplots were placed in paper bags and stored in a dark, ventilated environment until samples were completely desiccated. After taxonomic identification, specimens were inserted into labeled herbarium envelopes and deposited in the Bryophyte Section of the University of the Azores Herbarium (AZU). The collection is archived under the title: “MOVECLIM—AZORES Project: Bryophytes from Flores Island (2013)”.

The same plots were also surveyed for vascular plants following the GIMS protocol [53]. This procedure involves assessing each 10 m × 10 m, which is subdivided into four 5 m × 5 m subplots. All subplots are inventoried for vascular plant species, and each species is assigned a cover value based on Braun Blanquet Ordinal Transform Value (OTV) [69]. The source of the taxonomic nomenclature follows Silva et al. [70], updated by the Azorean Biodiversity Portal [31].

#### 4.1.4. Fieldwork Locations

**Figure 11:**
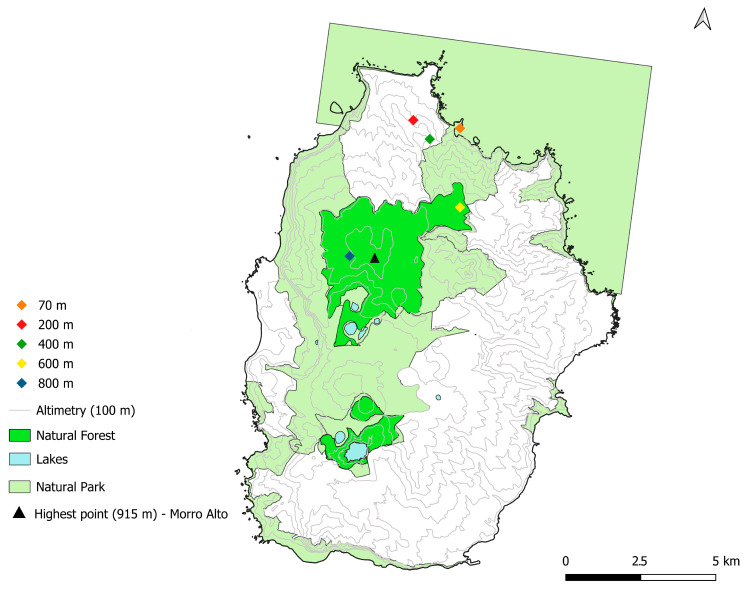
Distribution of bryophyte sampling sites across Flores Island (Azores), overlaid on topographic contours (100 m intervals). Sites are colored by locality (70 to 800 m a.s.l.). Areas shaded in light green represent the Flores Natural Park, in green, represent the natural forests, while blue polygons indicate lakes.

This study was conducted across five sites distributed along an elevational gradient on Flores Island (Azores, Portugal), including areas within the Flores Natural Park (Table 1, Figure 11). The sampling transects span from *Ponta do Ilhéu* at 70 m a.s.l. to *Morro Alto* at 800 m a.s.l., encompassing elevations of 70, 200, 400, 600, and 800 m a.s.l.

Below is a brief characterization of the vegetation at each bryophyte collection area.

**Figure 12:**
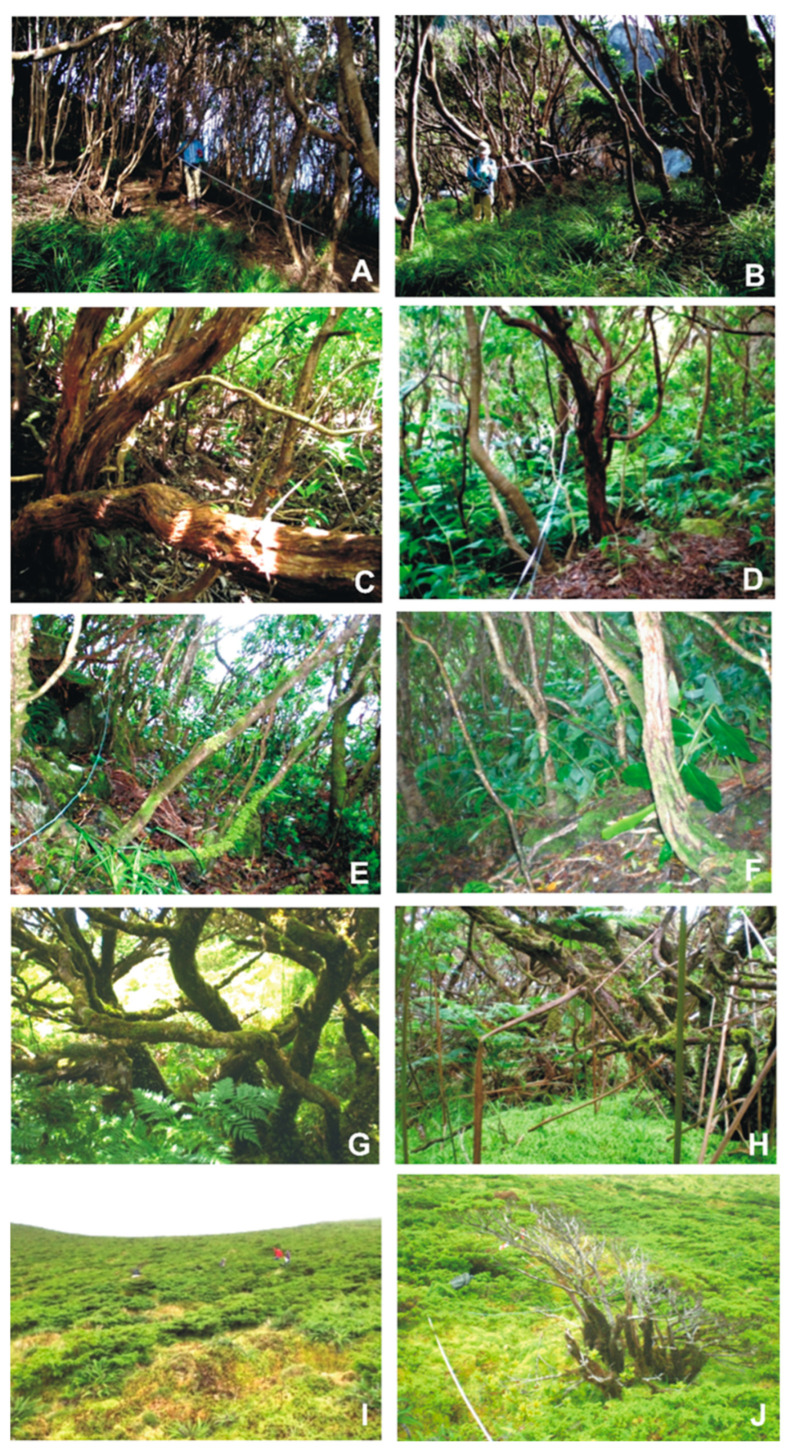
Overview of vegetation in the ten sampling plots used for the bryophyte survey on Flores Island, shown in pairs by locality and increasing elevation: (**A**,**B**) *Ponta do Ilhéu* (70 m); (**C**,**D**) *Caminho para Ponta Delgada* (200 m); (**E**,**F**) *Outeiros* (400 m); (**G**,**H**) *Ribeira do Cascalho* (600 m); (**I**,**J**) *Morro Alto* (800 m). Each locality is represented by two adjacent plots (1 and 2).

- *Ponta do Ilhéu* (70 m a.s.l.)—Vegetation at this lowland site is dominated by *Picconia azorica* (Tutin) Knobl. (Endemic), *Pittosporum undulatum* Vent. (Introduced/Invasive species), and *Morella faya* (Aiton) Wilbur. (Native). The forest canopy reaches an average height of 5.2 m. Bryophyte cover ranges from 5% to 25%, distributed across soil, rocks, and tree trunks (Figure 12A,B);
- *Caminho para Ponta Delgada* (200 m a.s.l.)—The forest includes species such as *Erica azorica* Hochst. ex Seub. (Endemic), *Vaccinium cylindraceum* Sm. (Endemic), *Juniperus brevifolia* (Hochst. ex Seub.) Antoine (Endemic), and *Morella faya*, alongside the invasive *Pittosporum undulatum*. The canopy reaches up to 5.8 m. Bryophytes are most abundant on rocky surfaces, with approximately 25% cover, and occur less frequently on soil and tree bark (Figure 12C,D);
- *Outeiros* (400 m a.s.l.)—This mid-elevation forest exhibits a higher canopy, reaching up to 6.2 m. However, approximately 50% of the vegetation is composed of *Pittosporum undulatum*, and large individuals of *Hedychium gardnerianum* Sheppard *ex* Ker-Gawl. (Introduced/Invasive species) are present. Native species such as *Erica azorica*, *Picconia azorica*, *Laurus azorica* (Seub.) Franco (Endemic), *Morella faya*, and *Vaccinium cylindraceum* occur with relative abundances ranging from 15% to 40%. Bryophyte cover is highest on tree trunks (~60%) but is also substantial on the forest floor (~40%) (Figure 12E,F);
- *Ribeira do Cascalho* (600 m)—This forested site, known locally as “Zimbral,” is dominated by *Juniperus brevifolia*, comprising approximately 90% of the canopy, which reaches a height of 3.9 m. Other species, including *Vaccinium cylindraceum* (30%), *Ilex azorica* Gand. (Endemic) (25%), *Laurus azorica* (15%), and *Myrsine retusa* Aiton (Endemic) (25%), are present at lower frequencies. Bryophyte cover is extensive, reaching approximately 80% on both soil and tree surfaces (Figure 12G,H);
- *Morro Alto* (800 m)—Located within a designated Nature Reserve, this site features high-altitude scrubland dominated by *Juniperus brevifolia* and *Calluna vulgaris* (L.) Hull (Native), with *Struthiopteris spicant* (L.) Weis (Native) also abundant. Vascular plants do not exceed 2 m in height. The bryophyte layer covers nearly 100% of the ground surface, predominantly composed of *Sphagnum* spp. Epiphytic bryophytes are also well represented, with average coverage reaching up to 30% (Figure 12I,J).

### 4.2. Study Taxa

#### 4.2.1. Bryophytes

Bryophytes are ancestral, small, rootless plants, comprising around 20,000 species worldwide, and include mosses, liverworts, and hornworts [53]. They exhibit diverse life strategies [71,72] that enable them to colonize a wide range of habitats in all terrestrial ecosystems, from coastal zones to forests and urban areas. These sensitive plants are highly dependent on their immediate environment, responding to various changes, including substrate composition and acidity, precipitation, temperature, salinity, and pollution [49].

These plants play crucial roles in ecosystems, such as retaining water and regulating the water cycle [73,74], and often acting as pioneers, creating favorable conditions for the establishment and development of non-pioneer organisms [75]. These groups are found in all terrestrial ecosystems, including coastal zones, natural forests, urban areas, and pastures, among others [76].

#### 4.2.2. Bryophytes in the Azores

The Azores, with approximately 475 *taxa* (308 mosses, 162 liverworts, and 5 hornworts) [30,31], rank among the three Atlantic archipelagos with the highest bryophyte species richness [77], which is a tribute to the high dispersion ability typical of this group. The Canary Islands, with about 617 *taxa* (six hornworts, 194 liverworts, and 417 mosses) [78], exhibit the greatest bryophyte diversity, followed by the Madeira archipelago, which contains approximately 546 *taxa* (six hornworts, 175 liverworts, and 365 mosses) [79]. The rate of endemism is relatively low on bryophytes, but presently seven moss species and three liverwort species are considered Azorean endemics [31,62,80].

### 4.3. Identification and Categorization of Specimens

#### 4.3.1. Taxonomic Framework

The nomenclature and taxonomy of the bryoflora on Flores Island are based on established taxonomic keys specific to this group of plants. Moss identification was largely based on the floras by Smith [81] and Casas et al. [82], while liverworts were identified using the works of Paton [83], Casas et al., [84], and the identification key developed by Schumacker &Váňa [85] for Europe. Visual guides [74,86,87], as well as online resources from the Azorean Biodiversity Portal [31], the British Bryological Society and the ‘*Bildatlas der Moose Deutschlands*’ were also consulted. Taxonomic nomenclature follows the framework established by Gabriel and colleagues [30], incorporating updates available through the Azorean Biodiversity Portal [31].

#### 4.3.2. Biogeographic Framework

The identified species have been classified into the following biogeographic categories: Azorean endemics, Macaronesian endemics, European endemics, and Native [30].

#### 4.3.3. Conservation Framework

The conservation status of the different species was assessed according to the categories of the IUCN Red List in Europe [32,88]: Not Evaluated (NE), Data Deficient (DD), Least Concern (LC), Near Threatened (NT), Vulnerable (VU), Endangered (EN), Critically Endangered (CR), Extinct in the Wild (EW), and Extinct (EX).

### 4.4. Sampling Completeness and Diversity Measurement

First-order Jackknife estimator was used to estimate the completeness of the bryophyte, moss, and liverwort inventories, as it performs well with presence-absence data and is particularly suited for datasets with many rare species [89].

Currently, one of the most accepted approaches for quantifying diversity from species abundance data is the use of effective numbers of species, also known as Hill numbers [90,91,92]. Hill numbers form a unified family of diversity indices that differ only in a single parameter, q, which determines their sensitivity to species relative abundances [90]. When *q* = 0, all species are weighted equally, regardless of their frequency; as *q* increases, common species receive progressively more weight, while rare species become less influential [90,91].

This formulation also places traditional indices into the same conceptual framework: species richness corresponds to *q* = *0*; Shannon entropy becomes comparable when exponentiated (*q* = *1*); and Simpson’s index corresponds to the inverse of the concentration index (*q* = *2*). In this way, values are expressed in consistent units (“effective number of species”), which makes comparisons across indices and communities more intuitive [90,91,92].

In practical terms, four orders of Hill numbers were used in this study: (a) species richness (S) (*q* = 0), the simple count of species at a site; (b) the exponential of Shannon–Wiener entropy (H’) (*q* = 1) [93]; (c) the inverse of Simpson’s concentration index (D) (*q* = 2); and (d) the inverse of Berger–Parker’s index (d) (*q* = ∞), which emphasizes dominance by the most abundant species [94]. Together, these indices integrate complementary aspects of diversity, reflecting richness, rarity, and dominance within the same framework [95,96].

In addition to alpha diversity, beta diversity was also assessed to evaluate the degree of compositional turnover among sites. In this context, the fewer the species shared between communities, the higher the beta diversity [97]. Pairwise similarity coefficients were applied to compare communities in both qualitative and quantitative terms.

Species composition similarity was also investigated using cluster analysis, both for elevation and the substrate. Hierarchical cluster analysis (UPGMA) was made based on species richness across five altitudinal levels and six substrates, using Bray–Curtis dissimilarity. In this method, the sites or substrates are joined based on the average distance between all members in the two groups. Values on the dendrogram scale represent Bray–Curtis dissimilarity (0 = identical composition; 1 = completely dissimilar).

All analyses were made using the free software Past 4.03 [98].

### 4.5. Altitudinal Gradient and Mid-Domain Effect Analysis

To test whether the altitudinal pattern of bryophyte richness could be explained by a mid-domain effect (MDE), we modeled the expected distribution of species richness using a null model based on random overlap of species’ altitudinal ranges. For each species, the observed minimum and maximum elevations were used to define its range size. We then simulated 10,000 randomizations in which the position of each range was reassigned randomly within the observed altitudinal domain (10–800 m a.s.l.), while keeping its size constant. For each simulation, we calculated species richness at the five sampled elevations (10, 200, 400, 600 and 800 m). The mean and 95% confidence intervals of the simulated richness values were compared with the observed richness. Agreement between the observed and predicted richness patterns was assessed visually and using a Spearman rank correlation test. All analyses were performed in R version 4.5.2 (R Core Team, Vienna, Austria), using the packages dplyr, ggplot2, and tidyverse.

## 5. Conclusions

This study bridges floristic and ecological approaches, integrating diversity metrics, endemism, and substrate preferences into a unified analysis. It demonstrates that bryophyte diversity on Flores Island is structured primarily by elevation, which integrates key climatic and ecological gradients. Species richness and evenness peak at mid-elevations, where moderate temperatures, high humidity, and structural complexity favor the coexistence of lowland and montane *taxa*. Substrate type further modulates community composition, reflecting the importance of local-scale habitat heterogeneity. Together, topography, substrate diversity, and vegetation complexity explain the observed distributional patterns and underscore the ecological distinctiveness of Flores’ bryophyte flora, revealing that strong community turnover can occur over remarkably short altitudinal ranges, emphasizing the ecological sensitivity of island bryophyte assemblages.

From a conservation perspective, these findings highlight the need to maintain elevational continuity and microhabitat diversity within the island’s native forests. Mid-elevation woodlands, particularly those retaining structural complexity and frequent cloud immersion, act as refugia for hygrophilous and endemic species. Because bryophyte assemblages respond rapidly to subtle climatic and structural changes, they serve as sensitive indicators of environmental disturbance and forest integrity. These altitudinal trends reflect not only contemporary climatic gradients but also the biogeographical and historical processes shaping bryophyte assemblages across the archipelago.

This integrative framework provides a baseline for future long-term monitoring and comparative studies across Macaronesian islands, providing valuable early-warning signals of climate-driven changes in species distributions and community structure. More broadly, the pronounced ecological differentiation over short spatial distances on Flores underscores both the fragility and the exceptional scientific value of island ecosystems as natural laboratories for understanding biodiversity responses to environmental change.

## Figures and Tables

**Table 1:** Geographic and topographic characteristics of the ten bryophyte sampling plots established on Flores Island (Azores). Each locality includes two plots, with values of elevation, slope, aspect (exposure), and geographic coordinates. Pairs of plots (P1 and P2) were established within each locality to capture within-site variability. Coordinates are in decimal degrees; exposure is expressed in azimuth degrees (0° = N).

Plot Code	Locality	Elevation (m a.s.l.)	Slope (Degrees)	Exposure (Degrees)	Latitude (Decimal °)	Longitude (Decimal °)
FLO_0070_P1	*Ponta do Ilhéu*	70	15	280	39.50633	−31.19453
FLO_0070_P2		77	18	107	39.50622	−31.19461
FLO_0200_P1	*Caminho para Ponta Delgada*	249	35	141	39.50689	−31.21286
FLO_0200_P2		266	32	14	39.50661	−31.21275
FLO_0400_P1	*Outeiros*	399	40	81	39.50192	−31.20558
FLO_0400_P2		399	41	54	39.50183	−31.20558
FLO_0600_P1	*Ribeira do* *Cascalho*	649	5	90	39.48281	−31.19042
FLO_0600_P2		648	8	176	39.48267	−31.19033
FLO_0800_P1	*Morro Alto*	833	14	278	39.46319	−31.22594
FLO_0800_P2		833	15	283	39.46325	−31.22600

## Data Availability

The full dataset used in this article, ‘MOVECLIM—AZORES project: Bryophytes from Flores Island (2013)’, is deposited in GBIF [28], a publicly available database. URL: https://doi.org/10.15468/943by2 (accessed on 22 October 2025). Release date: 5 June 2025.

## Supplementary Materials

The following supporting information can be downloaded at: https://www.mdpi.com/article/10.3390/plants14243766/s1, Table S1: Taxonomic classification of the bryoflora of Flores Island (Azores), including Phylum, Class, Order, Family, Scientific Name, Abbreviation (Scientific Name), Colonization Status, and IUCN Status.
